# Mining Public Toxicogenomic Data Reveals Insights and Challenges in Delineating Liver Steatosis Adverse Outcome Pathways

**DOI:** 10.3389/fgene.2019.01007

**Published:** 2019-10-18

**Authors:** Mohamed Diwan M. AbdulHameed, Venkat R. Pannala, Anders Wallqvist

**Affiliations:** ^1^Department of Defense Biotechnology High Performance Computing Software Applications Institute, Telemedicine and Advanced Technology Research Center, U.S. Army Medical Research and Development Command, Fort Detrick, MD, United States; ^2^The Henry M. Jackson Foundation for the Advancement of Military Medicine, Inc., Bethesda, MD, United States

**Keywords:** liver steatosis, adverse outcome pathway, MIE, steatosis AOP, data mining

## Abstract

Exposure to chemicals contributes to the development and progression of fatty liver, or steatosis, a process characterized by abnormal accumulation of lipids within liver cells. However, lack of knowledge on how chemicals cause steatosis has prevented any large-scale assessment of the 80,000+ chemicals in current use. To address this gap, we mined a large, publicly available toxicogenomic dataset associated with 18 known steatogenic chemicals to assess responses across assays (*in vitro* and *in vivo*) and species (i.e., rats and humans). We identified genes that were differentially expressed (DEGs) in rat *in vivo*, rat *in vitro*, and human *in vitro* studies in which rats or *in vitro* primary cell lines were exposed to the chemicals at different doses and durations. Using these DEGs, we performed pathway enrichment analysis, analyzed the molecular initiating events (MIEs) of the steatosis adverse outcome pathway (AOP), and predicted metabolite changes using metabolic network analysis. Genes indicative of oxidative stress were among the DEGs most frequently observed in the rat *in vivo* studies. *Nox4*, a pro-fibrotic gene, was down-regulated across these chemical exposure conditions. We identified eight genes (*Cyp1a1*, *Egr1*, *Ccnb1*, *Gdf15*, *Cdk1*, *Pdk4*, *Ccna2*, and *Ns5atp9*) and one pathway (retinol metabolism), associated with steatogenic chemicals and whose response was conserved across the three *in vitro* and *in vivo* systems. Similarly, we found the predicted metabolite changes, such as increases of saturated and unsaturated fatty acids, conserved across the three systems. Analysis of the target genes associated with the MIEs of the current steatosis AOP did not provide a clear association between these 18 chemicals and the MIEs, underlining the multi-factorial nature of this disease. Notably, our overall analysis implicated mitochondrial toxicity as an important and overlooked MIE for chemical-induced steatosis. The integrated toxicogenomics approach to identify genes, pathways, and metabolites based on known steatogenic chemicals, provide an important mean to assess development of AOPs and gauging the relevance of new testing strategies.

## Introduction

Liver steatosis, which is characterized by excessive accumulation of lipids (i.e., mainly triglycerides) in hepatocytes (Tiniakos et al., 2010), is a widely prevalent liver disease affecting more than a quarter of the world’s population (McPherson et al., 2015; Younossi et al., 2018). It is a progressive disease that can lead to more severe forms, such as steatohepatitis, fibrosis, cirrhosis, hepatocellular carcinoma, and ultimately, liver failure (McPherson et al., 2015). A number of other adverse health effects, such as diabetes, metabolic syndrome, and cardiovascular disease, are associated with this disease (Ballestri et al., 2016; Mikolasevic et al., 2016; Younossi et al., 2018). Steatosis occurs in both alcoholic and non-alcoholic fatty liver disease (AFLD/NAFLD) (Toshikuni et al., 2014). The latter of which has been attributed to unhealthy diet and a sedentary lifestyle (Tiniakos et al., 2010). Recent studies show that chemical exposures contribute to steatosis development and progression (Cave et al., 2011; Schwingel et al., 2011; Kaiser et al., 2012; Al-Eryani et al., 2015). For example, healthy, non-alcoholic workers in a vinyl chloride factory were found to have steatohepatitis comparable to that of alcoholic liver disease patients (Cave et al., 2010). Such chemical exposure-induced steatosis, known as toxicant-associated fatty liver disease (TAFLD), is a sub-class of NAFLD (Al-Eryani et al., 2015).

Currently, there are no therapeutics available for treating steatosis. In light of this circumstance, reducing or avoiding exposures to steatogenic chemicals (i.e., chemicals that cause steatosis) is considered the best approach to prevent or treat chemical-induced steatosis. This requires that we screen and identify steatogenic chemicals. However, current animal-based (*in vivo*) testing approaches are time consuming and allow us to study only a few chemicals at a time. Not surprisingly, the steatosis-causing potential of the more than 80,000 chemicals produced throughout the world is largely unknown. Hence, there is a need to develop alternative *in vitro* assays for identifying steatogenic chemicals. This, in turn, requires a detailed understanding of the molecular mediators and key pathways associated with this steatosis.

**Figure 1:**
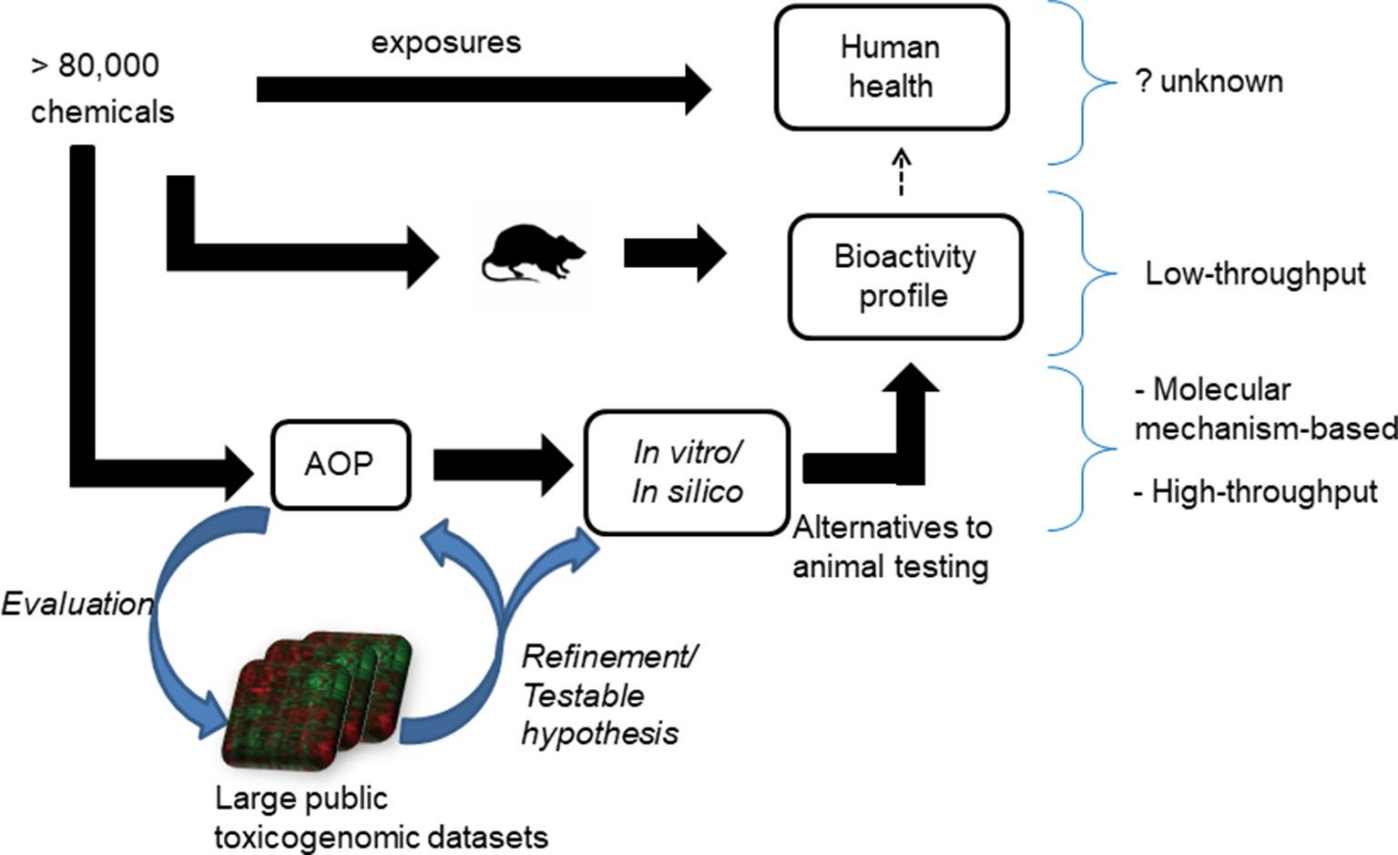
Current approaches to understand the adverse health effects associated with chemical exposures. The effect of more than 80,000 chemicals on human health is unknown. Animal studies can be used to create bioactivity profile of chemicals and make inference on their effect on human health, but the sheer size of the number of chemicals needed to be tested precludes a comprehensive survey. A proposed alternative is to use adverse outcome pathways (AOPs) to generate mechanistic *in vitro*/*in silico* tests as alternatives to animal tests of toxicity. The mining of public toxicogenomic datasets plays a role in helping to evaluate and refine AOPs.

The adverse outcome pathway (AOP) framework is a recent development in toxicology that helps us to understand disease processes. This framework summarizes the current knowledge of chemical-induced disease processes from molecular- to phenotype-level changes and aids in the development of mechanism-based alternative testing approaches (Ankley et al., 2010; Oki et al., 2016). It typically captures the essence of a complex disease phenotype in a simple flowchart-like diagram comprising molecular initiating events (MIEs), key events (KEs), and adverse outcomes (Vinken, 2013). This type of causal, mechanistic outline allows for the development of *in vitro* tests for MIEs/KEs, which potentially capture the adverse outcome observed in *in vivo* studies (Figure 1). One successful application of the AOP framework is the development of *in vitro* skin sensitization tests, which are now part of the guidelines provided by regulatory agencies, as alternatives to animal-based testing approaches (Kleinstreuer et al., 2018). This success can be attributed to the ability of the AOP framework to capture the key molecular events *in vivo*, reproduce them under *in vitro* conditions, develop *in vitro* tests focused on those KEs, and validate them using large chemical datasets.

In the case of liver steatosis, a putative AOP has been proposed (Mellor et al., 2016). It lists 10 ligand-activated transcription factors (predominantly nuclear receptors) as MIEs and triglyceride accumulation as the KE (**Supplementary Figure S1**). The proposed MIEs are the liver x receptor (LXR), aryl hydrocarbon receptor (AhR), pregnane x receptor (PXR), peroxisome proliferator-activated receptor (PPAR)-γ, PPAR-α, farnesoid x receptor (FXR), constitutive androstane receptor (CAR), retinoic acid receptor (RAR), glucocorticoid receptor (GR), and estrogen receptor (ER) (Mellor et al., 2016). These MIEs are postulated to affect four major physiological events in the liver, namely lipid entry, *de novo* lipid synthesis, lipid metabolism through β-oxidation, and lipid efflux (Angrish et al., 2016). For this putative AOP to be practically useful, each MIE and KE event must be experimentally proven to be relevant for a diverse set of steatogenic chemicals. However, most experimental studies have focused on only a few exemplar steatogenic chemicals, such as amiodarone, tetracycline, and valproic acid, or a subset of genes (Sahini et al., 2014; Szalowska et al., 2014; Vitins et al., 2014). The MIEs/KEs should also be evaluated in terms of whether they are conserved across assays (*in vivo* and *in vitro*) and whether the results from animal studies will be relevant for humans. The validity of the AOP as well as whether the identified key components hold true across diverse chemicals, assay/test systems, and species, needs to be evaluated. In this context, computational data mining of large publicly available toxicogenomic datasets offers a way to evaluate whether the AOP components hold true across a diverse set of chemicals and allow us to identify molecular level mediators that are conserved across both assay systems (e.g., *in vivo* and *in vitro*) and species (**Figure 1**).

In this work, we addressed two main questions: *1*) what molecular-level mediators (genes, metabolites), pathways, and MIEs are conserved among rat *in vivo* exposure studies for a diverse set of steatogenic chemicals, and *2*) which of these molecules, signals, and events are conserved across rat and human *in vitro* experiments? To this end, we performed computational data mining/toxicogenomic analyses of a large publicly available toxicogenomics database, Toxicogenomics Project-Genomics Assisted Toxicity Evaluation (TG-GATEs) (Igarashi et al., 2015). This database includes rat *in vivo*, rat *in vitro*, and human *in vitro* studies of exposure to chemicals at different doses and times. This allowed us to analyze and compare toxicogenomic data across both assays (*in vivo* and *in vitro*) and species using a diverse set of steatogenic chemicals. Using the parallelogram analysis approach (Kienhuis et al., 2009), the molecular mediators and pathways found to be common across the three test systems are expected to be relevant to human exposures (Sutter, 1995). We focused our analyses on known steatogenic chemicals that are part of TG-GATEs.

Specifically, we performed toxicogenomics-based parallelogram analysis of 18 diverse steatogenic chemicals and identified eight differentially expressed genes that were conserved across the three test systems: *Cyp1a1*, *Egr1*, *Ccnb1*, *Gdf15*, *Cdk1*, *Pdk4*, *Ccna2*, and *Ns5atp9*. *Nox4*, a pro-fibrotic gene, was down-regulated across these chemical exposure conditions. The retinol metabolism pathway was a significantly enriched pathway frequently conserved across the three test systems. Analysis of MIEs in the current steatosis AOP showed that nuclear receptor-mediated lipogenesis activation was not observed for the current set of steatogenic chemicals and highlights that mitochondrial toxicity can be considered as one of steatosis MIE. Metabolic network analysis shows overlap of 41 predicted metabolites across the three test systems. Overall, our toxicogenomics-based parallelogram analysis of diverse steatogenic chemicals enabled us to identify steatosis-relevant genes, pathways, and metabolites conserved across three test systems. Our work addresses the current AOP for steatosis, identifies knowledge gaps, and suggests ways to improve it and aid the development of new screening tools.

## Methods

### Dataset and Pre-Processing of Data

We used TG-GATEs, a large publicly available toxicogenomics dataset (Igarashi et al., 2015) that includes gene expression data obtained from subjects given a treatment (i.e., exposure to a diverse set of chemicals at different doses and time points) and matched controls. An important feature of this dataset is that it includes rat *in vitro*, rat *in vivo*, and human *in vitro* data. We identified eighteen chemicals represented in TG-GATEs and reported in the literature to produce steatosis (Sahini et al., 2014; McDyre et al., 2018): amiodarone, amitriptyline, bromobenzene, carbon tetrachloride, colchicine, coumarin, diltiazem, disulfiram, ethanol, ethionamide, ethinyl estradiol, hydroxyzine, imipramine, lomustine, puromycin aminonucleoside, tetracycline, vitamin A, and valproic acid (**Supplementary Table S1**). We downloaded the raw CEL files from the TG-GATEs website (https://toxico.nibiohn.go.jp/open-tggates/english/search.html, accessed in July 2017) and carried out separate pre-processing for the three test systems (rat *in vivo*, rat *in vitro*, and human *in vitro* assays) using the protocol described in our previous studies (AbdulHameed et al., 2014; AbdulHameed et al., 2016). Briefly, we performed quantile normalization of the raw CEL files using the robust multi-array average (RMA) method in the BioConductor/R package *affy* (Gautier et al., 2004; Gentleman et al., 2004). We then carried out quality assessment using the *ArrayQualityMetrics* package and removed outlier arrays (Kauffmann et al., 2009). We re-processed the remaining arrays using the RMA method and used the resulting data for further analyses. We used the *MAS5calls* function in *affy* and removed probe sets that were “absent” in all replicates across all chemical exposures. We then carried out non-specific filtering of genes using the *genefilter* package and removed probe sets with no Entrez ID or low variance across chemical exposures (Gentleman et al., 2018). We calculated the average log_2_ intensity between replicates of a chemical exposure condition (i.e., exposure to a particular chemical at a particular dose and time point), and log-ratios for each gene between treatment conditions (groups) and their corresponding control conditions (groups).

### Differential Gene Expression Analysis

To identify DEGs, we used the rank product method (Breitling et al., 2004), which has found widespread use in gene expression studies (de Tayrac et al., 2011; Goonesekere et al., 2014). The method employs a non-parametric approach to convert fold-change values into ranks and calculates the statistical significance of the obtained ranks. We used the Bioconductor/R package *RankProd* for this analysis (Del Carratore et al., 2017). Each chemical exposure condition was subjected to rank product analysis and up- or down-regulated genes with a false discovery rate cut-off of 0.05 were identified as DEGs for that exposure condition.

To compare common DEGs between rat *in vitro* or *in vivo* and human *in vitro* exposure conditions, we used the *biomaRt* package and mapped human gene IDs to rat gene IDs (Durinck et al., 2009). To facilitate comparisons for the set of 18 steatogenic chemicals, we focused on the most frequently observed genes, pathways, and metabolites. We considered a set of genes, pathways, and metabolites to be frequent if it was commonly observed in 5% of the exposure conditions.

### Pathway Enrichment Analysis

We conducted Kyoto Encyclopedia of Genes and Genomes (KEGG) pathway (Kanehisa et al., 2017) enrichment analysis using the *enrichKEGG* function in the BioConductor/R package *clusterProfiler* (Yu et al., 2012). This function performs enrichment analysis using the hypergeometric test to identify significant pathways. We carried out pathway enrichment analysis using the DEGs associated with each chemical exposure condition. We used Cytoscape to summarize enriched pathways for each chemical as a chemical-pathway network (Shannon et al., 2003).

### Analysis of Activation of Molecular Initiating Events

The MIEs in the proposed steatosis AOP are ligand-activated transcription factors and regulate the expression of their target genes. Activation of such MIEs will lead to altered (either increased or decreased) expression of their target genes, which can be detected in gene expression data. By analyzing the expression levels of genes targeted by these MIEs, it would be possible to provide measures of their activation by different steatogenic chemicals. To this end, we collected existing transcription factor-target gene data from the Transcriptional Regulatory Relationships Unraveled by Sentence-based Text mining (TRRUST) database (Han et al., 2015). In addition to the nine MIEs whose activation or agonism were linked to steatosis, two key genes downstream of them, SREBP1c (gene symbol: *Srebf1*) and ChREBP (gene symbol: *Mlxipl*), are also Transcription Factors (TFs). We included them in this analysis, and refer to the nine MIE genes and two key target genes together as MIEs in this work. Nine of 11 MIEs mapped to TRRUST database.

### Metabolic Network Analysis

We used the reconciled rat (*iRno*) and human (*iHsa*) genome-scale metabolic network reconstructions (GENREs) developed by our group (Blais et al., 2017) to predict toxicant-specific metabolite alterations in plasma. We recently updated the rat genome-scale metabolic model by modifying some of the existing reactions based on evidence in the literature (Pannala et al., 2018). In the current work, we generated an updated version of the human metabolic network by reconciling the changes that were incorporated into the rat network (**Supplementary Table S2**). We validated these models by testing their ability to successfully simulate 327 liver-specific metabolite functions that represented liver metabolism. We have provided the updated rat model as part of the original publication (Pannala et al., 2018) and the updated human model in this study (**Supplementary Table S3**).

To simulate the metabolite predictions for the 18 steatogenic chemicals from the TG-GATEs database, we first determined the appropriate boundary conditions under which the experimental data were generated (Igarashi et al., 2015). For *in vitro* studies, we used the uptake rates determined for the hepatocyte studies reported in our original publication (Blais et al., 2017). For the rat *in vivo* studies, we used the liver uptake rates obtained from satiated rats in a previous study (Orman et al., 2013).

We used the Transcriptionally Inferred Metabolic Biomarker Response (TIMBR) algorithm to predict toxicant-induced perturbations in metabolites by integrating gene expression changes with GENREs (Blais et al., 2017). Briefly, TIMBR converts the log_2_ fold-change (log_2_FC) values of all DEGs into weights (*W*) for each of the gene-protein-reaction (GPR) relationships in the GENRE. These reaction weights are then transformed into larger (or smaller) weights to represent relative levels of expression between the control and toxicant-treated conditions. TIMBR then calculates the global network demand required to produce a metabolite (*X* *_met_*) as follows, by minimizing the weighted sum of fluxes across all reactions for each condition and metabolite, so as to satisfy the associated mass balance and an optimal fraction of the maximum network capability (*v* *_opt_*) to produce a metabolite.

(1) $$\begin{array}{l} X_{\mathrm{met}}=\min\sum W\cdot |v|\\ s.\;t.:v_{x}\geq v_{\mathrm{opt}};v_{\mathrm{lb}}<v{<}_{\mathrm{ub}};S\cdot v=0 \end{array}$$

Here, *W* denotes the vector representing the reaction weights, *v* is a vector of reaction fluxes, and *S* is the stoichiometric matrix (see Blais et al., 2017 for details). We incorporated the aforementioned boundary conditions for uptake and secretion rates into the algorithm by fixing the respective lower (*v* *_lb_*) and upper bounds (*v* *_ub_*) of the exchangeable reactions (*v* *_ex_*) in the model (Eq. 2).

(2) $$v_{\mathrm{lb}}<v_{\mathrm{ex}}<v_{\mathrm{ub}}$$

Using this method, we determined the relative production scores for all metabolites (*X* *_raw_*) from control (*X* *_control_*) and toxicant-treated (*X* *_treatment_*) conditions (Eq. 3), and then calculated the TIMBR production scores (*X* *_s_*) as the z-transformed scores across all exchangeable metabolites (Eq. 4).

(3) $$X_{\text{raw}}=\frac{X_{\text{control}}-X_{\text{treatment}}}{X_{\text{control}}+X_{\text{treatment}}}$$

(4) $$X_{S}=\frac{X_{\text{raw}}-\mu }{\sigma }$$

Here, the model predictions of altered metabolite levels were considered as having increased or decreased in the blood based on TIMBR production score cut-off values of greater than 0.1 or less than −0.1, respectively. Metabolites with scores between −0.1 and 0.1 were considered as unchanged.

## Results

### Rat *in Vivo* Gene Expression Analysis

#### DEGs

We analyzed data for 18 steatogenic chemicals represented in the TG-GATEs database, collected from rats exposed to these chemicals, and identified significantly modulated DEGs across all available dose and duration conditions. Pre-processing of the raw gene expression data resulted in a log_2_FC matrix with 205 exposure conditions and 7,272 genes (**Supplementary Table S4**). Of the 7,272 genes, 1,423 were differentially expressed in at least one of the exposure conditions. **Supplementary Figure S2** shows the effects of dose and duration of exposure on the number of DEGs. Of the 205 exposure conditions, 100 (∼50%) showed less than 50 DEGs, reflecting the sparsity of DEGs (i.e., most exposure conditions showed few DEGs). We observed a dose-dependent effect in which compared to low doses, high doses were associated with more DEGs. Exposure to ethionamide, ethinyl estradiol, puromycin aminonucleoside, lomustine, or amiodarone showed more than 200 DEGs. In contrast, exposure to vitamin A, ethanol, or tetracycline showed less than 50 DEGs (**Supplementary Table S5**).

#### Most Frequent DEGs

**Figure 2:**
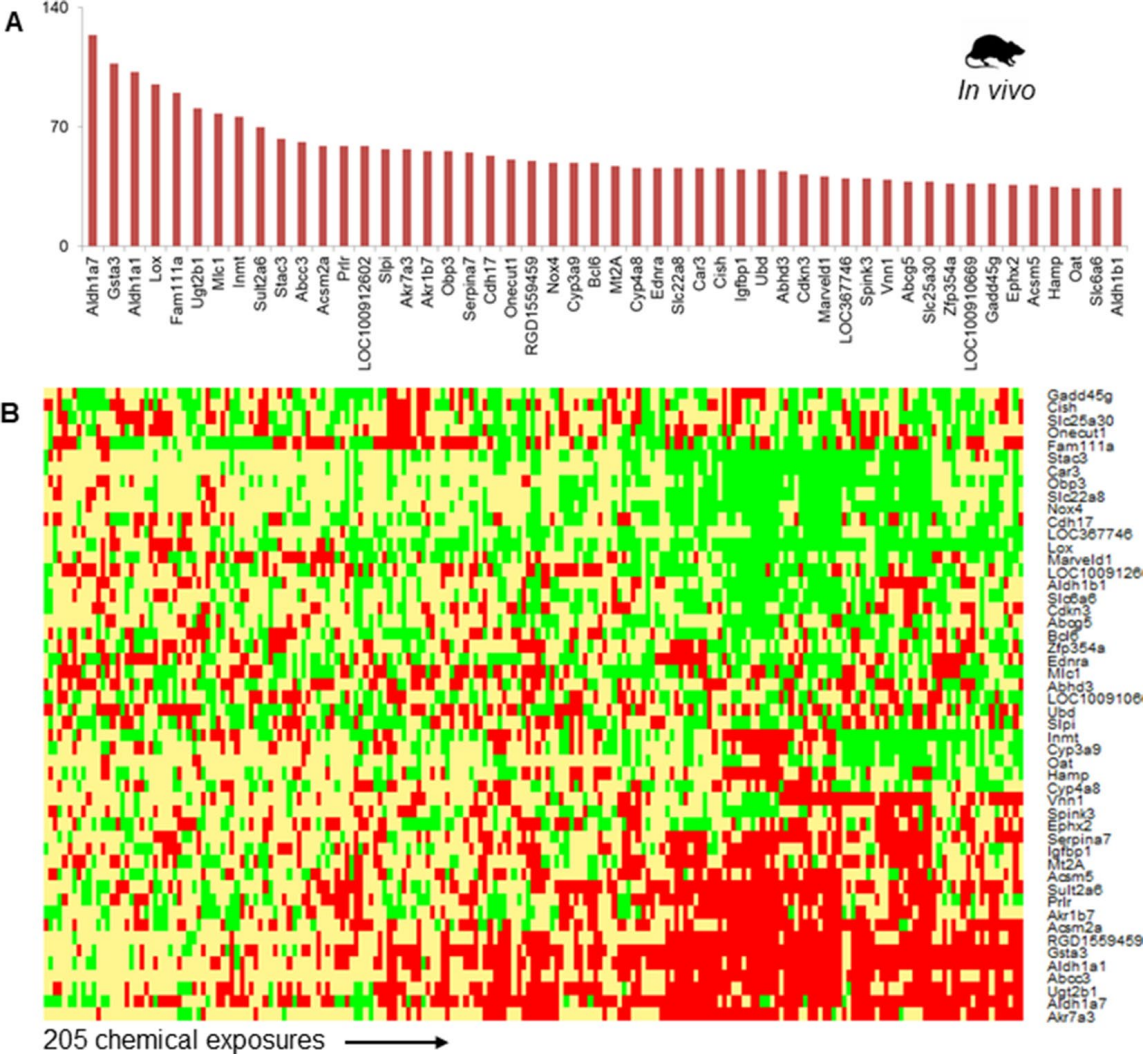
**(A)** The 50 most frequently observed differentially expressed genes (DEGs) in rats exposed to steatogenic chemicals *in vivo*. **(B)** Heat map of log_2_fold-change (log_2_FC) values associated with the top 50 DEGs across 205 steatogenic chemical exposures. Red indicates up-regulation (> 0.6), yellow indicates lack of modulation (0.6 to -0.6), and green indicates down-regulation (< -0.6).

**Figure 2A** shows the top 50 frequently occurring DEGs across the 205 chemical exposure conditions. *Aldh1a7*, *Gsta3*, and *Aldh1a1* were the most frequently observed DEGs, being differentially expressed in more than 100 chemical exposure conditions. The list of frequent DEGs contained genes from diverse families, including oxidoreductase enzymes (e.g., *Aldh1a7*, *Aldh1a1*, *Lox*, and *Nox4*), mitochondrial enzymes involved in fatty acid metabolism (e.g., *Acsm2a* and *Acsm5*), transporters (e.g., *Abcc3*, *Slc22a8*, and *Abcg5*), enzymes involved in xenobiotic metabolism (e.g., *Cyp3a9*, *Sult2a6,* and *Ugt2b1*), metal binding protein *Mt2A*, and other enzymes (e.g., *Car3* and *Ephx2*). **Figure 2B** shows a heatmap of the fold-change values for the top 50 most frequent DEGs. Whereas genes such as *Akr7a3*, *Aldh1a7*, *Ugt2b1*, *Abcc3*, *Gsta3*, *Acsm2a*, and *Acsm5* were up-regulated, genes such as *Stac3*, *Car3*, *Nox4*, and *Lox* were down-regulated (**Figure 2B**).

Phase-II metabolizing enzymes, such as *Sult2a6* and *Ugt2b1*, were up-regulated across most steatogenic exposure conditions. Two mitochondrial enzymes, *Acsm2a* and *Acsm5* were up-regulated and among the top 50 frequent DEGs (**Figure 2A**). We found that *Nox4* was down-regulated for most exposure conditions (17 of 18 steatogenic chemicals). For example, for the exemplar steatogenic chemical, amiodarone, *Nox4*, expression was down-regulated (log_2_FC < -0.6) at all high-dose exposure conditions.

#### KEGG Pathway Enrichment Analysis

We identified 84 pathways that were significantly enriched for at least one of the chemicals in rat *in vivo* studies. Of the 205 exposure conditions, 114 had at least one significantly enriched KEGG pathway with more than two DEGs mapping to that pathway. The retinol metabolism pathway, associated with 92 exposure conditions, was the most frequently enriched pathway. Xenobiotic metabolism-related pathways, steroid hormone biosynthesis, and bile secretion pathways were the other frequently enriched pathways (i.e., enriched in more than 25 exposure conditions) (**Supplementary Figure S4**). With respect to particular chemicals, diltiazem, amiodarone, amitriptyline, colchicine, disulfiram, and ethonamide showed more than 15 enriched pathways.

**Figure 3:**
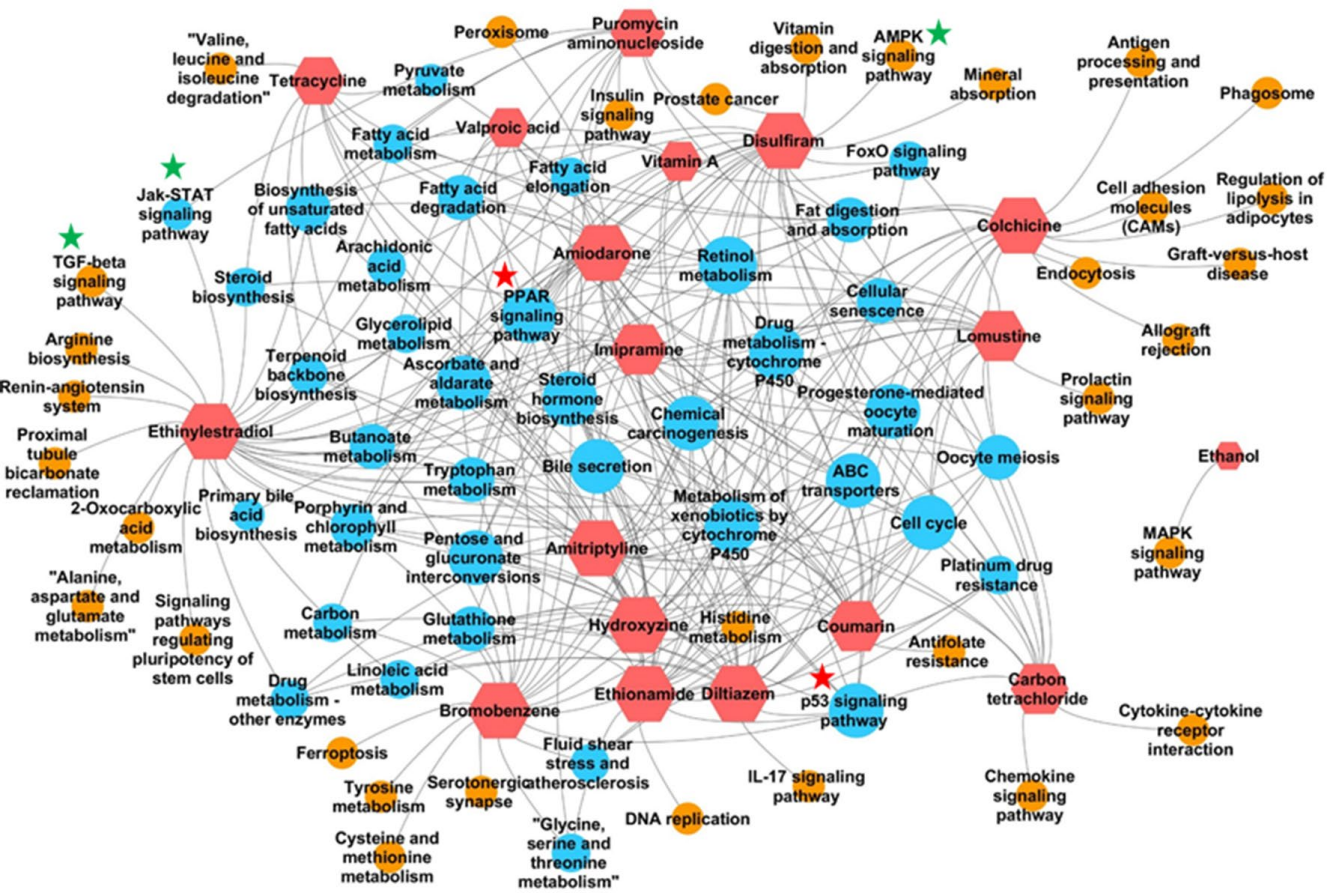
Pathways enriched for each chemical. Red hexagons indicate steatogenic chemicals. Blue circles indicate pathways enriched for two or more chemicals. Orange circles indicate pathways enriched for only one chemical. An edge (shown in grey) was created between the chemical and the pathway if the pathway is enriched in any dose/time exposures for that particular chemical. Signaling pathways enriched for more than two chemicals were marked with red start. Signaling pathways enriched for one or two chemicals were marked with green star.

In addition to identifying commonly enriched pathways for steatogenic chemicals, we also analyzed the pathways that were associated with specific chemicals. The presence of chemical-specific steatosis-relevant pathway enrichment would suggest the presence of diverse mechanisms leading to steatosis. As such, we risk overlooking chemical-specific mechanisms by focusing on common pathways. To address this concern, we created a chemical-pathway matrix (18 chemicals vs. 84 enriched pathways) in which a pathway is linked to a chemical if it is enriched for that chemical at any dose or duration of exposure. **Figure 3** summarizes the chemical-pathway matrix as a network, where pathways unique to a particular chemical are shown as orange circles and those enriched in two or more chemicals are shown as blue circles. The retinol metabolism pathway was enriched for 15 chemicals, except for puromycin aminonucleoside, carbon tetrachloride, and ethanol. Other signaling pathways, such as Jak-STAT, TGF-beta, and AMPK, were enriched for mostly one but in some cases two steatogenic chemicals (**Figure 3**, green stars).

#### Analysis of Steatosis Molecular Initiating Event (MIE) Activation

The MIEs in the proposed steatosis AOP are ligand-activated TFs and are predominantly nuclear receptors. A characteristic feature shared by these MIEs is that upon being activated by ligand binding, they modulate the expression of their target genes. This feature motivated us to analyze the expression pattern of these target genes and use it as an indicator of MIE activation. Similar approach of TF-target gene information were used to analyze gene expression data and identify TF modulators (Ryan et al., 2016). We collected the target genes associated with these 11 MIEs from the TRRUST database and then mapped them to the pre-processed gene expression dataset (i.e., 7,272 genes). PPARγ and ESR1 had the highest numbers of target genes mapped (45 and 44, respectively). None of the *Nr1i3* (CAR) genes mapped to the pre-processed data. *Nr1h3*, *Nr1h2*, and *Mlxipl* showed the fewest mapped genes.

We calculated how many target genes were differentially expressed for each steatogenic chemical (at any dose or duration) (**Table 1**). None of the chemicals significantly modulated more than ∼40% of the MIE target genes. The number of differentially expressed target genes of *Ahr* was highest for disulfiram [5 of 13 genes (38%)], followed by ethinyl estradiol and puromycin aminonucleoside [4 of 13 genes (31%)]. The MIE that showed differentially expressed target genes for the most steatogenic chemicals was *Nr1i2* (PXR). For seven chemicals (amiodarone, carbon tetrachloride, coumarin, diltiazem, ethinyl estradiol, hydroxyzine, and lomustine), 4 of 15 (27%) *Nr1i2* target genes showed differential expression.

**Table 1:** Target genes differentially expressed for each molecular initiating event (MIE).^a^

Rat *in vivo*	AHR	ESR1	NR1H4	NR1I2	NR3C1	PPARG	RARA	SREBF1
*nMAPPED*	*13*	*40*	*13*	*15*	*22*	*45*	*16*	*20*
Amiodarone	2 (15)	4 (10)	2 (15)	**4 (27)**	4 (18)	2 (4)	**4 (25)**	4 (20)
Amitriptyline	2 (15)	5 (13)	2 (15)	3 (20)	0 (0)	2 (4)	2 (13)	1 (5)
Bromobenzene	2 (15)	5 (13)	2 (15)	1 (7)	2 (9)	3 (7)	2 (13)	0 (0)
Carbon tetrachloride	2 (15)	4 (10)	**4 (31)**	**4 (27)**	3 (14)	4 (9)	**4 (25)**	2 (10)
Colchicine	1 (8)	2 (5)	1 (8)	1 (7)	0 (0)	2 (4)	1 (6)	1 (5)
Coumarin	3 (23)	6 (15)	2 (15)	**4 (27)**	0 (0)	2 (4)	2 (13)	3 (15)
Diltiazem	1 (8)	3 (8)	2 (15)	**4 (27)**	0 (0)	1 (2)	2 (13)	0 (0)
Disulfiram	**5 (38)**	8 (20)	2 (15)	2 (13)	2 (9)	5 (11)	**4 (25)**	**5 (25)**
Ethanol	1 (8)	3 (8)	1 (8)	0 (0)	1 (5)	2 (4)	1 (6)	2 (10)
Ethinyl estradiol	**4 (31)**	6 (15)	1 (8)	**4 (27)**	3 (14)	9 (20)	9 (31)	5 (25)
Ethionamide	3 (23)	5 (13)	3 (23)	3 (20)	3 (14)	3 (7)	2 (13)	5 (25)
Hydroxyzine	3 (23)	7 (18)	1 (8)	4 (27)	1 (5)	1 (2)	2 (13)	2 (10)
Imipramine	2 (15)	5 (13)	2 (15)	3 (20)	2 (9)	2 (4)	3 (19)	3 (15)
Lomustine	2 (15)	9 (23)	2 (15)	**4 (27)**	2 (9)	9 (20)	3 (19)	4 (20)
Puromycin aminonucleoside	**4 (31)**	8 (20)	3 (23)	3 (20)	3 (14)	7 (16)	3 (19)	**5 (25)**
Tetracycline	0 (0)	0(0)	1 (8)	0 (0)	0 (0)	0 (0)	0 (0)	1 (5)
Valproic acid	1 (8)	1(3)	1 (8)	0 (0)	1 (5)	1 (2)	0 (0)	0 (0)
Vitamin A	0 (0)	0 (0)	0 (0)	0 (0)	0 (0)	0 (0)	1 (6)	2 (10)

a Only MIEs with more than 10 target genes are shown. Each cell shows the number of target genes differentially expressed, with the percentage of target genes differentially expressed in parentheses. If >20% of MIE target genes were differentially expressed, the number of target genes is marked in bold.

#### Metabolic Network Analysis

Using a genome-scale metabolic model for rat, we carried out metabolic network analyses by integrating gene expression data to predict alterations in metabolites, focusing on a subset of 119 lipid-related metabolites. Clustering of the predicted metabolite modulations across the 205 chemical exposure conditions resulted in three distinct metabolite and condition clusters. *Metabolite cluster 1* (**Supplementary Figure S4**), which included saturated fatty acids (e.g., palmitate and stearate), unsaturated fatty acids (e.g., myristic and valeric acid), glycolipids, sphingosine derivatives, and phosphatidylcholine derivatives, had the most altered metabolite profiles and was selected as the cluster most relevant for the 18 steatogenic chemicals. It should be noted that this consistently altered metabolites group was obtained directly in an unsupervised manner. Our analysis predicted the metabolites to be increased for the *Conditions cluster C1* and decreased in *Condition cluster C2*. *Condition cluster C1* consisted of 24% high-dose, 35% medium-dose, and 41% low-dose conditions, whereas *Condition cluster C2* consisted of 45% high-dose, 25% medium-dose, and 22% low-dose conditions.

### Rat *in Vitro* Analysis

Pre-processing of rat *in vitro* data for the 18 steatogenic chemicals resulted in a log_2_FC matrix with 7,362 genes and 152 chemical exposure conditions, of which 123 showed at least one DEG. Among the 7,362 genes in the pre-processed set, 1,201 were differentially expressed for at least one chemical. **Supplementary Figure S5A** shows the 50 DEGs most frequently observed across the 152 exposure conditions. *Cyp1a1* was the most common DEG (31 exposure conditions), followed by *Cyp26b1* (29 exposure conditions) and *Gdf15* (22 exposure conditions). Pathway enrichment analysis of each exposure condition revealed that 42 of the 152 conditions showed at least one enriched pathway and that the most frequently enriched pathways were the interleukin (IL)-17 signaling, TNF signaling, and retinol metabolism pathways (> 10 exposure conditions). Our metabolic network analysis and subsequent clustering of the predicted lipid-related metabolite (119) profiles across the 152 exposure conditions identified a metabolite cluster of 54 metabolites that showed alterations across most exposure conditions. We selected this metabolite cluster, which included a diverse group of lipids (e.g., palmitate, stearate, oleate, glycolipids, and phosphatidylcholines), as the cluster relevant to the steatogenic chemicals.

### Human *in Vitro* Analysis

We pre-processed the human *in vitro* data and obtained a log_2_FC matrix with 10,158 genes and 97 exposure conditions. These data included exposure conditions associated with 16 of the 18 steatogenic chemicals (data for ethanol and puromycin aminonucleoside were lacking). Of the 97 exposure conditions, 77 showed at least one DEG. The number of DEGs was highest for colchicine, valproic acid, and diltiazem (> 90), whereas it was lowest for imipramine (< 5). Among the 10,158 genes in the pre-processed set, 898 were differentially expressed for at least one chemical. **Supplementary Figure S5B** shows the 50 DEGs most frequently observed across the 97 exposure conditions. *CYP1A1* was the most common DEG (17 exposure conditions). Pathway enrichment analysis showed that 22 of the 97 exposure conditions showed at least one enriched pathway and that the most frequently enriched pathways were those of retinol metabolism and chemical carcinogenesis (10 exposure conditions). A metabolic network analysis and subsequent clustering of the predicted lipid-related metabolite (119) profiles across the 97 exposure conditions identified a metabolite cluster of 43 metabolites that showed alterations across most exposure conditions. This cluster, which we selected as the cluster relevant to the steatogenic chemicals, included saturated and unsaturated fatty acids, glycolipids, and phosphatidylcholines, as in the case of the rat *in vivo-in vitro* data.

### Parallelogram Analysis

Parallelogram analysis is an approach to identify common mechanisms and molecular mediators underlying gene expression data obtained from *in vivo* studies and *in vitro* assays, or those obtained from different species. The genes and pathways common to the three systems examined here (rat *in vivo*, rat *in vitro*, and human *in vitro*) are assumed to be relevant to living humans. To facilitate comparisons for the set of 18 steatogenic chemicals, we focused on the most frequently observed genes, pathways, and metabolites.

**Figure 4:**
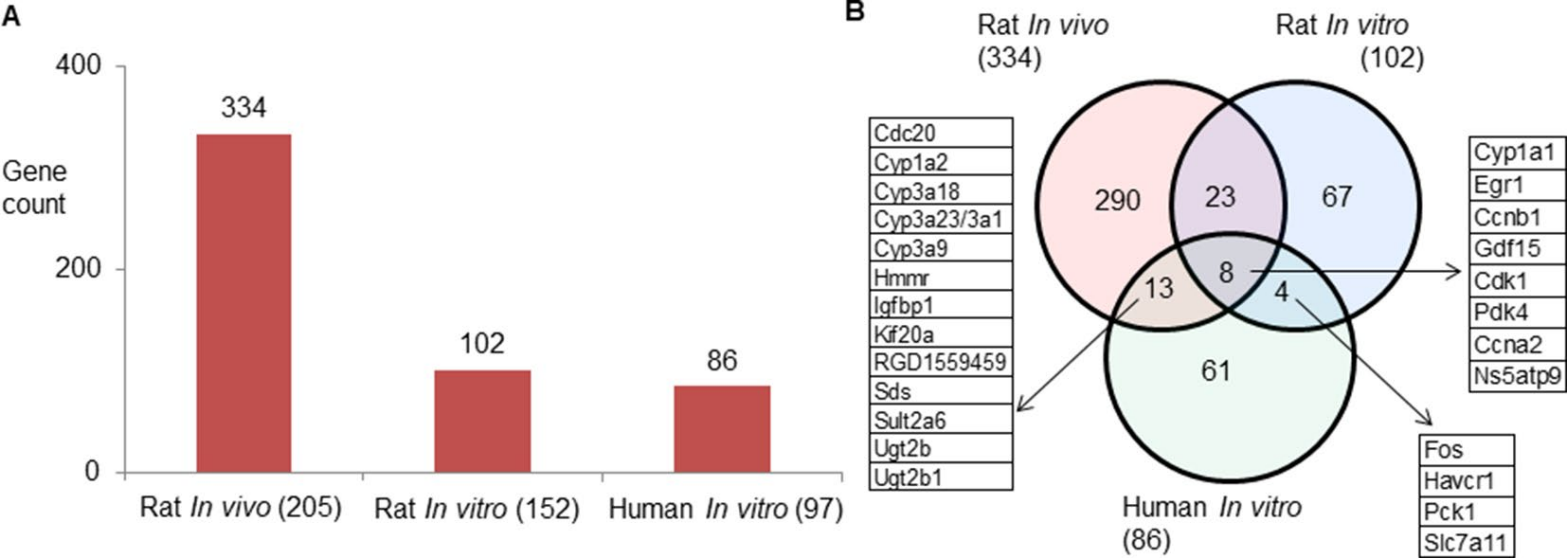
**(A)** Count of frequently differentially expressed genes in three test systems (rat *in vivo*/*vitro* and human *in vitro*). The total number of exposures in each test system is given in parenthesis. **(B)** Genes commonly expressed differentially across test systems. Eight genes were commonly differentially expressed in three test systems.

**Figure 5:**
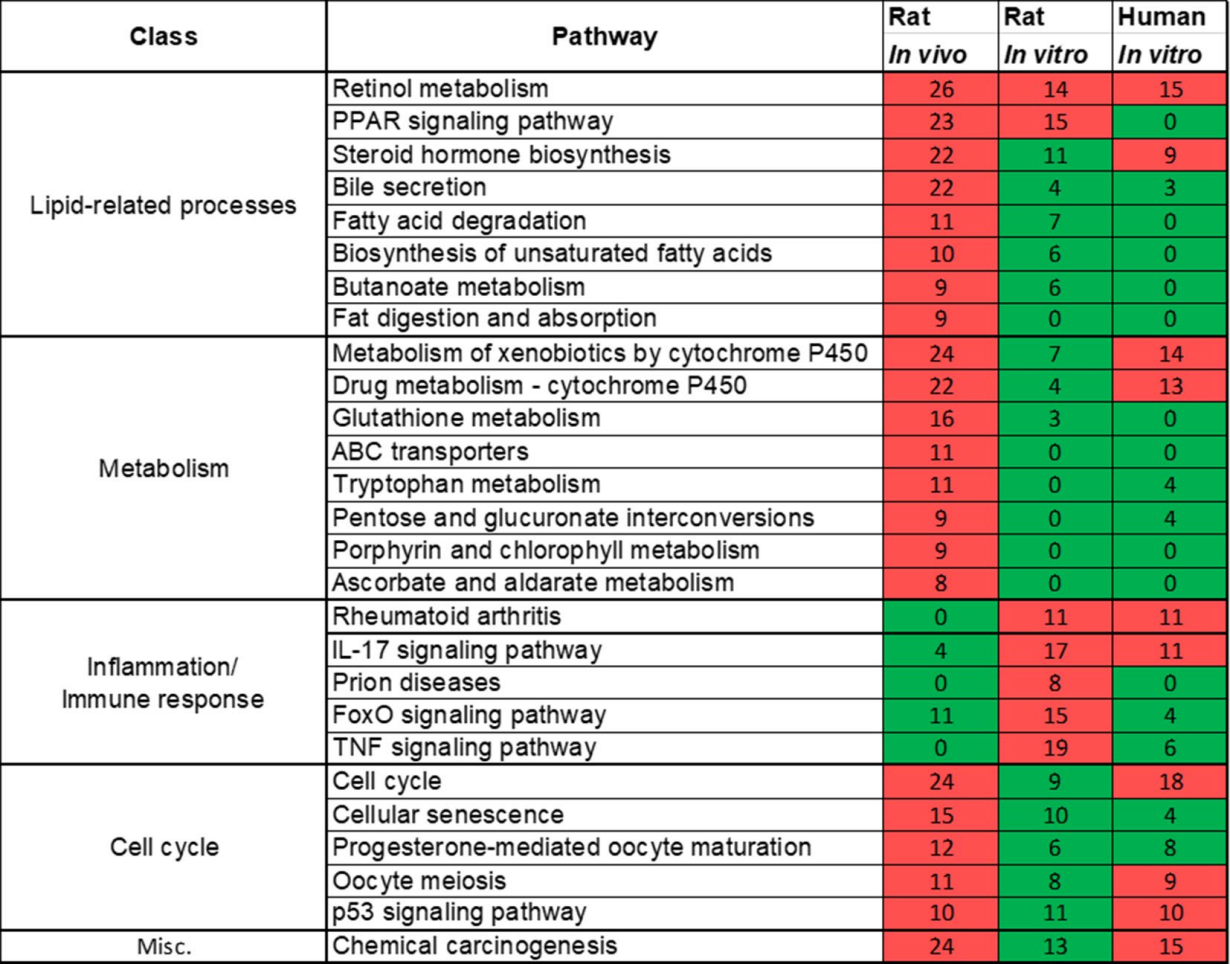
Pathways commonly enriched across three test systems (rat *in vivo*/*vitro* and human *in vitro*). A significantly enriched pathways is colored in red, otherwise it is colored in green. The number indicates the count of differentially expressed genes that mapped in that pathway. If a gene is differentially expressed for any chemical exposure and mapped to this pathway, they were counted together. Retinol metabolism pathway was commonly enriched in all three test systems.

**Figure 6:**
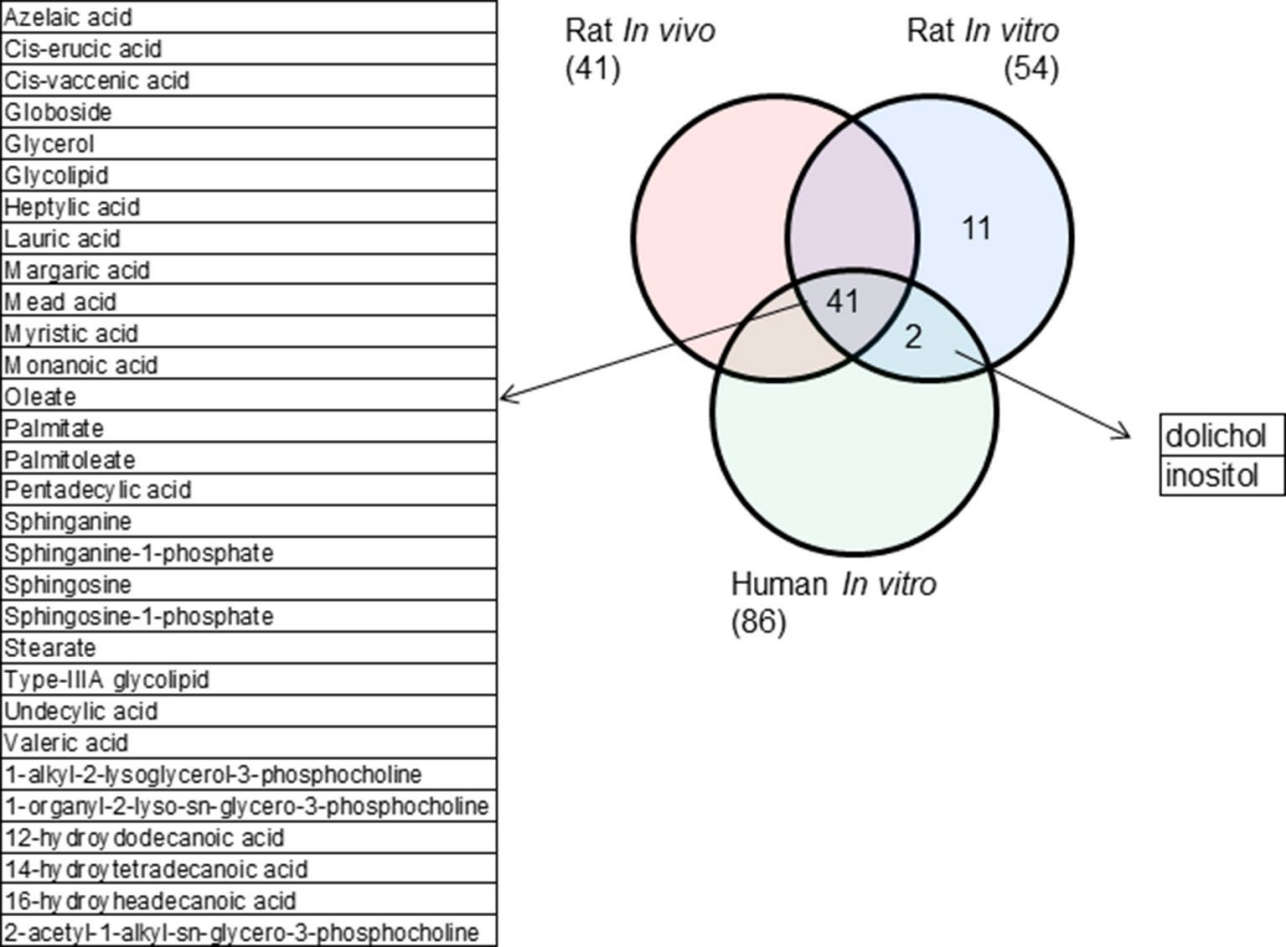
Metabolites commonly modulated across the three test systems.

Rat *in vivo*, rat *in vitro*, and human *in vitro* showed 334, 102, and 86 frequently observed DEGs, respectively, across all steatogenic chemical exposure conditions (**Figure 4A**). **Figure 4B** shows the overlap of these DEGs between the three systems. Rat *in vivo*-rat *in vitro* overlap (31 DEGs) was higher than the rat *in vivo*-human *in vitro* overlap (21 DEGs). The overlap was smallest between rat *in vitro* and human *in vitro* systems (12 DEGs). The following eight DEGs were found frequently in all three systems: *Cyp1a1*, *Egr1*, *Ccnb1*, *Gdf15*, *Cdk1*, *Pdk4*, *Ccna2*, and *Ns5atp9*. **Figure 5** shows the overlap of pathways across the three test systems. The retinol metabolism pathway was enriched in all three test systems. The PPAR signaling pathway was enriched in the rodent test systems but not in human *in vitro* system. **Figure 6** shows the overlap of metabolites across the three systems. Forty-one of the predicted metabolites were commonly mapped to all three systems. All metabolites predicted in the rat *in vivo* system were also observed in both *in vitro* systems. The rat *in vitro* system predicted 11 metabolites that were not modulated in the other two systems, whereas the human *in vitro* and rat *in vitro* systems shared two metabolites in common.

## Discussion

In this study, we performed computational data mining of public toxicogenomics dataset to show its utility in evaluating/refining steatosis adverse outcome pathway (AOP). An important distinction of this work is that we have analyzed the whole genome level expression changes associated with a large, diverse set of 18 steatogenic chemicals at different dose and time points in rat *in vivo*/*in vitro* and human *in vitro* assays with the goal of finding molecular level mediators and pathways associated with chemical-induced steatosis.

Mellor et al. previously proposed ligand-activated transcription factors (predominantly nuclear receptors) as MIEs in liver steatosis AOP (Mellor et al., 2016). On the other hand, Kaiser and colleagues identified mitochondrial toxicity as the major mechanism associated with steatogenic chemicals in IRIS (Integrated Risk Information System), the U.S. Environmental Protection Agency’s toxicity database (Kaiser et al., 2012). Many of the exemplar steatogenic chemicals, such as amiodarone, bromobenzene, carbon tetrachloride, tetracycline, and valproic acid, are known to cause mitochondrial toxicity, which leads to oxidative stress development (Wong et al., 2000; Begriche et al., 2011; Kaiser et al., 2012).

In our **analysis of most frequent DEGs**, we find that genes associated with response to oxidative stress/lipid peroxidation were most frequently differentially expressed, i.e., conserved for the diverse set of steatogenic chemicals. For example, among the most frequent DEGs are the aldehyde dehydrogenases (ALDHs), aldo-keto reductases (AKRs), and glutathione-s-transferases (GSTs) (**Figure 2A**). Mitochondrial toxicity is known to increase ROS levels, which subsequently increases lipid-peroxidation (Ucar et al., 2013) and leads to the formation of large numbers (> 200) of highly reactive aldehyde compounds (Singh et al., 2013) which are cytotoxic. ALDHs such as *Aldh1a7* and *Aldh1a1* are enzymes that protect cells from aldehyde-induced cytotoxicity by oxidizing aldehydes to acids (Singh et al., 2013). At high stress levels other enzymes belonging to the class of aldo-keto reductase detoxify aldehydes by converting them to alcohol (Ayala et al., 2014). Among the top-50 DEGs, we found two AKRs (*Akr7a3* and *Akr1b7*). Enzymes involved in xenobiotic metabolism, as well as transporters which participate in elimination of toxic compounds from cells, such as *Cyp3a9*, *Sult2a6*, *Ugt2b1*, *Abcc3*, *Abcg5*, and *Slc22a8*, were also among the most frequent DEGs. Overall, our analysis of the most frequent DEGs across diverse steatogenic chemicals agrees with occurrence of mitochondrial toxicity. In contrast, we did not find genes involved in *de novo* fatty acid synthesis or transport among the top-most frequent DEGs, which is contradictory to what we would expect from previously proposed steatosis AOP. *Fasn* is a key determinant enzyme in *de novo* fatty acid synthesis and is known to be up-regulated in human steatosis (Dorn et al., 2010). We find that this gene was differentially expressed in only 14 of the 205 chemical exposures. Similar to our observation reported here, previous study of tetracycline exposures in mouse precision-cut liver slices did not observe up-regulated expression of lipogenic genes (Szalowska et al., 2014).

Another notable gene in the most frequent DEG list is *Nox4*, which plays a key role in the development of liver fibrosis (Crosas-Molist and Fabregat, 2015). Impairment of NOX4 expression in HepG2 cells was also associated with an anti-apoptotic mechanism (Caja et al., 2009). We found that *Nox4* was down-regulated for most exposure conditions (17 of 18 steatogenic chemicals), an effect which could reflect a compensatory response to reduce the prevailing oxidative stress. For example, for the exemplar steatogenic chemical, amiodarone, *Nox4* expression was down-regulated (log_2_FC < -0.6) at all high-dose exposure conditions. This is an interesting new finding, as down-regulation of *Nox4* upon exposure to a steatogenic chemical like amiodarone has not been reported previously. Further experiments are needed to test whether this down-regulation represents a tipping point between steatosis and steatohepatitis (i.e., steatosis associated with severe inflammation). To evaluate the potential of NOX4 as possible biomarker, future experiments could also quantify the rate, magnitude, and reversibility of NOX4 expression during and after withdrawal of steatogenic chemical exposure (Blaauboer et al., 2012).

In our **analysis of frequently enriched pathways**, we found the retinol metabolism pathway to be the most frequently enriched pathway for 18 steatogenic chemicals (**Supplementary Figure 3**). Alteration of this pathway has also been reported in mice and NAFLD patients (Vitins et al., 2014; Pettinelli et al., 2018). Interestingly, we found the same pathway to be frequently enriched for a diverse class of steatogenic chemicals. Mapping of frequent DEGs showed that genes involved in metabolism (e.g., *Cyp3a9, Cyp4a8, Cyp1a1, Cyp1a2, Ugt2b*, and *Ugt2b1*), ALDHs (e.g., *Adh4, Aldh1a1*, and *Aldh1a7*), and retinol saturase (*retsat*) mapped to this pathway (**Supplementary Figure 6**). The PPAR signaling pathway is another frequently enriched pathway. It is an important regulator of lipid homeostasis (Mellor et al., 2016). Although it is interesting to find lipogenesis-related genes mapped to this pathway, analysis of the fold-change values associated with these genes (such as *Fasn*, *Fads2*, and *Scd1*) do not show a clear relationship such as chemical exposure-induced up-regulation (**Supplementary Table S6**). For example, *Scd1* was down-regulated in high dose/time exposures for 8 of 18 chemicals and unchanged in the remaining 10 chemicals. This result is contrary to the previous report of increased expression of *Fads2* and *Scd1* in NASH patients (Chiappini et al., 2017) and could represent differences associated with chemical-induced steatosis vs general NAFLD. Another gene, *Me1*, was up-regulated in high dose/time exposures of 12 of 18 chemicals (**Supplementary Table S6**). *Me1* plays a key role in lipogenesis by contributing NADPH to fatty acid synthesis. However, *Me1* is also known to play a protective role by contributing NADPH under oxidative stress/high ROS environment (Gorrini et al., 2013). We suggest that the observed up-regulation of *Me1* represents response to oxidative stress rather than lipogenesis. We did not find previous reports on up-regulation of *Me1* after steatogenic chemical exposures. In addition, we analyzed pathways enriched for one or few chemicals (**Figure 3**). We found TGF-β and AMPK signaling pathway to be associated with ethinyl estradiol and disulfiram exposures, respectively. Both of these pathways were known to be associated with NAFLD (Yang et al., 2014; Garcia et al., 2019). This highlights the multifactorial nature of steatosis and the possibility of a chemical utilizing more than one mechanism to induce it.

We evaluated the **activation of the MIEs** in the previously proposed steatosis AOP and found that 60% of the target genes were not differentially expressed by any of the 18 steatogenic chemicals. Most of the mapped/differentially expressed target genes were related to xenobiotic metabolism, transport, or cell cycle. For example, high numbers of AhR and PXR target genes were modulated by the greatest number of steatogenic chemicals (**Table 1**). These two targets play a major role as xenobiotic sensors. In our study, *Cyp3a9*, a key target gene of PXR that is up-regulated upon PXR is activation, was actually down-regulated for high dose/time exposures of amitriptyline, bromobenzene, carbon tetrachloride, coumarin, and puromycin aminonucleoside. In contrast, *Cyp1a1*, a key target gene of AhR, was up-regulated, indicating that AhR was activated. The enrichment of the retinol metabolism pathway also agrees with AhR activation, suggesting activation of AhR-mediated immune response (Mascolo et al., 2018). Previous studies show that AhR as well as PXR induces liver steatosis through induction of *CD36*, a fatty acid transporter (Zhou et al., 2008; Kawano et al., 2010; Lee et al., 2010). However, we did not observe differential expression of *CD36* gene. These findings suggest that activation of the target genes of a single nuclear receptor (e.g., AhR) cannot be used to causally link the receptor to steatosis unless a particular event (e.g., CD36 up-regulation) occurs. *Srebf1* is an important transcription factor that initiates *de novo* lipid synthesis and is implicated in NAFLD (Anderson and Borlak, 2008). Only four chemicals modulated 5 of the 20 *Srebf1* target genes, suggesting that up-regulation of lipogenesis might not be the initiating factor associated with most of the steatogenic chemicals studied here. Of note, exemplar toxicants such as amiodarone, tetracycline, and valproic acid modulates 4, 1, and 0 of *Srebf1* target genes, respectively. As mentioned earlier, *Fasn*, the key lipogenesis target gene of *Srebf1* was differentially expressed in only 14 of the 205 chemical exposures. Benet et al. reported that key genes of *de novo* fatty acid synthesis such as *Fasn* was not induced by 25 steatogenic drugs in HepG2 cells (Benet et al., 2014). They also report that SREBP1C (the human equivalent to rat *Srebf1*) was repressed by the steatogenic drugs (Benet et al., 2014). As these nuclear receptors participate in multiple cellular homeostatic functions, further experimental validation will be needed to quantify the direct relationship between nuclear receptor activation and steatosis. A key point that is highlighted by our MIE activation analysis is that because the activation of a nuclear receptor alone may not provide a causal link to the steatosis, it is also necessary to quantify the activation of additionally required molecular events.

Overall, our **steatosis MIE analysis**, performed by mining of public toxicogenomics database suggests mitochondrial toxicity rather than nuclear receptor activation as a possible MIE for chemical-induced steatosis. Additional reports in the literature suggest the involvement of nuclear receptors in steatosis, although they also show that such nuclear receptor interaction alone do not necessarily lead to steatosis (Sanyal et al., 2010; Neuschwander-Tetri et al., 2015; Cave et al., 2016). A recent large scale human clinical study, called as PIVENS, with 247 adults found that pioglitazone, a PPAR-γ agonist, significantly reduce steatosis (Sanyal et al., 2010) contrary to what we would expect if PPAR- γ agonism is a MIE for steatosis. Similarly, another human clinical trial found that a FXR agonist, reduce steatosis (Neuschwander-Tetri et al., 2015). This result contradicts the role of FXR agonism as a MIE for steatosis. FXR activation was reported to decrease *SREBP1C* gene and increase PPAR-α gene leading to decrease in lipid synthesis and increase in β-oxidation (Cave et al., 2016). This essentially shows that FXR activation has anti-steatotic effect rather than a MIE for steatosis. Multiple animal studies also reports that FXR agonism has anti-steatotic effect (Cipriani et al., 2010; Shen et al., 2011; Kunne et al., 2014). It should be noted that the above reports clearly highlight the relevance/involvement of nuclear receptors in steatosis. We only wish to emphasize that unless further experimental studies are carried out to dissect the links/relationship between nuclear receptor activation and steatosis formation, it may be inappropriate to consider certain nuclear receptors (e.g., FXR) in the current steatosis AOP as causal MIEs. CAR another MIE in the previously proposed AOP, was reported to repress the target genes of LXR, a key gene whose activation is known to up-regulate lipid synthesis (Cave et al., 2016). Our MIE activation analysis along with previous literature reports shows that further propagation/application of the previously proposed steatosis AOP should be used with caution, in particular, if attempts were made to develop individual quantitative structure-activity relationship (QSAR) models for each of these nuclear receptor MIEs and aggregate them to predict steatosis phenotype as explored by Gadaleta et al. (Gadaleta et al., 2018).

Utilizing mitochondrial toxicity as a potential MIE for steatosis is also consistent with the known toxic effects of the well-studied steatosis-causing chemicals amiodarone, valproic acid, and tetracycline. Agricultural and pharmaceutical chemicals are known to cause mitochondrial toxicity. There are standardized *in vitro* tests, including high-throughput screening assays for mitochondrial toxicity (Wills, 2017). This fact can be utilized to develop rapid screens for steatogenic chemicals and could be of interest to alternate test development groups. It should be noted that the current work is a computational data mining study that identifies a potential MIE for steatosis. It serves as a starting point to develop a steatosis AOP using mitochondrial toxicity as the MIE. However, this effort should include a detailed weight-of-evidence analysis, which is beyond the scope of the current analysis but should be part of future work. Drier et al. has summarized key events and other AOPs associated with mitochondrial toxicity (Dreier et al., 2019).

Finally, using **parallelogram analysis**, we examined the extent of conservation of the molecular-level mediators and pathways among *in vivo* and *in vitro* rat, and human *in vitro* experiments in TG-GATEs. Our overlap analysis showed that across all 3 exposure-study systems, 8 differentially expressed genes (*Cyp1a1*, *Egr1*, *Ccnb1*, *Gdf15*, *Cdk1*, *Pdk4*, *Ccna2*, and *Ns5atp9*), 1 pathway (retinol metabolism), and 41 predicted metabolite changes were commonly mapped. Although limited in scope, we can hypothesize based on the parallelogram approach that such mediators could be relevant for human exposures.

## Conclusion

Overall, our analysis of steatosis-causing chemicals in rat *in vivo* exposure studies pointed to oxidative stress as a major component of the disease etiology. Further, we suggest that in developing *in silico* (QSAR) models for predicting chemical-induced steatosis, previously proposed nuclear-receptor-based MIEs for steatosis should be used with caution, because *in vitro* binding/activation of the nuclear receptors may not translate into a steatosis-inducing signal given the complexity of their signaling mechanisms and cross-talk regulation. Instead, reflecting the multifactorial nature of the disease, mitochondrial toxicity has clear causal links to steatosis and should be considered as an additional MIE for chemical-induced steatosis.

The commonality in response to these chemicals between the *in vivo* and *in vitro* rat studies and the *in vitro* human studies occurred both on the gene and pathway level as well for the predicted metabolite changes. Based on the parallelogram approach, this commonality indicated a high likelihood for conservation and relevance of these events in human exposures. Overall, our analysis shows the utility of computational data mining of public toxicogenomics datasets to evaluate proposed steatosis AOP, suggests ways to improve it, identifies steatosis-relevant genes, pathways, and metabolites conserved across three test systems, and aids the development of new screening tools.

## Data Availability Statement

The data analyzed in this study was downloaded as raw CEL files from the TG-GATEs website (ftp://ftp.biosciencedbc.jp/archive, accessed in July 2017). All datasets generated for this study are included in the manuscript/**Supplementary Files**.

## Author Contributions

Conceived and designed the experiments: MA, VP, and AW. Analyzed the data: MA and VP. Contributed to the writing of the manuscript: MA, VP, and AW.

## Conflict of Interest

The authors declare that the research was conducted in the absence of any commercial or financial relationships that could be construed as a potential conflict of interest

## Supplementary Material

Supplementary Table S1 List of 18 steatogenic chemicals used in this study, along with their CAS numbers and molecular properties.

Supplementary Table S2 Details of the modifications made to the iHsa model.

Supplementary Table S3 Updated human genome-scale metabolic network model.

Supplementary Table S4 Log2fold-change matrix with 205 exposure conditions.

Supplementary Table S5 Maximum number of DEGs associated with each chemical exposure across the three test systems.

Supplementary Table S6 Log2fold-change values for genes Fasn, Fads2, Scd1, and Me1 under 205 exposure conditions.
