# Supplementary material for: Mining Public Toxicogenomic Data Reveals Insights and Challenges in Delineating Liver Steatosis Adverse Outcome Pathways
Source: Front Genet. 2019 Oct 18;10:1007. doi: 10.3389/fgene.2019.01007 (PMC6813744; doi:10.3389/fgene.2019.01007)
Supplement: Supplementary Table S1 — List of 18 steatogenic chemicals used in this study, along with their CAS numbers and molecular properties. [file Table_1.docx]

Supplementary Material

Mining public toxicogenomic data reveals insights and challenges in delineating liver steatosis adverse outcome pathways

Mohamed Diwan M. AbdulHameed,^1,2*^ Venkat R. Pannala,^1,2^ and Anders Wallqvist^2*^

*** Correspondence:** Mohamed Diwan M. AbdulHameed: mabdulhameed@bhsai.org; Anders Wallqvist: sven.a.wallqvist.civ@mail.mil

Supplementary Table S1. List of 18 steatogenic chemicals used in this study along with their CAS number and molecular properties.

| No | Steatogenic chemicals | CAS number | Molecular weight | XLogP |
| --- | --- | --- | --- | --- |
| 1 | Amiodarone | 1951-25-3 | 645 | 7.6 |
| 2 | Amitriptyline | 50-48-6 | 277 | 5 |
| 3 | Bromobenzene | 108-86-1 | 157 | 3 |
| 4 | Carbon tetrachloride | 56-23-5 | 154 | 2.8 |
| 5 | Colchicine | 64-86-8 | 399 | 1 |
| 6 | Coumarin | 91-64-5 | 146 | 1.4 |
| 7 | Diltiazem | 42399-41-7 | 415 | 3.1 |
| 8 | Disulfiram | 97-77-8 | 297 | 3.9 |
| 9 | Ethanol | 64-17-5 | 46 | -0.1 |
| 10 | Ethinylestradiol | 57-63-6 | 296 | 3.7 |
| 11 | Ethionamide | 536-33-4 | 166 | 1.1 |
| 12 | Hydroxyzine | 68-88-2 | 375 | 3.7 |
| 13 | Imipramine | 50-49-7 | 280 | 4.8 |
| 14 | Lomustine | 13010-47-4 | 234 | 2.8 |
| 15 | Puromycin aminonucleoside | 58-60-6 | 294 | -0.5 |
| 16 | Tetracycline | 60-54-8 | 444 | -2 |
| 17 | Valproic acid | 99-66-1 | 144 | 2.8 |
| 18 | Vitamin A | 68-26-8 | 286 | 5.7 |
