# Supplementary material for: Mining Public Toxicogenomic Data Reveals Insights and Challenges in Delineating Liver Steatosis Adverse Outcome Pathways
Source: Front Genet. 2019 Oct 18;10:1007. doi: 10.3389/fgene.2019.01007 (PMC6813744; doi:10.3389/fgene.2019.01007)
Supplement: Supplementary Table S5 — Maximum number of DEGs associated with each chemical exposure across the three test systems. [file Table_5.docx]

Supplementary Material

Mining public toxicogenomic data reveals insights and challenges in delineating liver steatosis adverse outcome pathways

Mohamed Diwan M. AbdulHameed,^1,2*^ Venkat R. Pannala,^1,2^ and Anders Wallqvist^2*^

*** Correspondence:** Mohamed Diwan M. AbdulHameed: mabdulhameed@bhsai.org; Anders Wallqvist: sven.a.wallqvist.civ@mail.mil

Supplementary Table S5. Maximum number of DEGs associated with each chemical exposures across the three test systems.

| Chemical exposure | Rat *in vivo* | Rat *in vitro* | Human *in vitro* |
| --- | --- | --- | --- |
| Amiodarone | 212 | 5 | 21 |
| Amitriptyline | 180 | 77 | 23 |
| Bromobenzene | 107 | 23 | 7 |
| Carbon tetrachloride | 187 | 77 | 14 |
| Colchicine | 74 | 88 | 91 |
| Coumarin | 137 | 61 | 33 |
| Diltiazem | 85 | 85 | 102 |
| Disulfiram | 182 | 75 | 80 |
| Ethanol | 36 | 57 | N/A |
| Ethinylestradiol | 269 | 21 | 8 |
| Ethionamide | 275 | 48 | 69 |
| Hydroxyzine | 108 | 84 | 88 |
| Imipramine | 150 | 82 | 1 |
| Lomustine | 213 | 37 | 81 |
| Puromycin aminonucleoside | 247 | 81 | N/A |
| Tetracycline | 33 | 20 | 36 |
| Valproic acid | 64 | 85 | 96 |
| Vitamin A | 45 | 38 | 61 |
