# Supplementary material for: Mining Public Toxicogenomic Data Reveals Insights and Challenges in Delineating Liver Steatosis Adverse Outcome Pathways
Source: Front Genet. 2019 Oct 18;10:1007. doi: 10.3389/fgene.2019.01007 (PMC6813744; doi:10.3389/fgene.2019.01007)
Supplement: Supplementary file 7 [file Presentation_1.pdf]

Supplementary Material

Mining public toxicogenomic data reveals insights and challenges in delineating liver steatosis adverse outcome pathways

Mohamed Diwan M. AbdulHameed,[1,2*] Venkat R. Pannala,[1,2] and Anders Wallqvist[2*]

*** Correspondence:** Mohamed Diwan M. AbdulHameed: mabdulhameed@bhsai.org; Anders Wallqvist: sven.a.wallqvist.civ@mail.mil

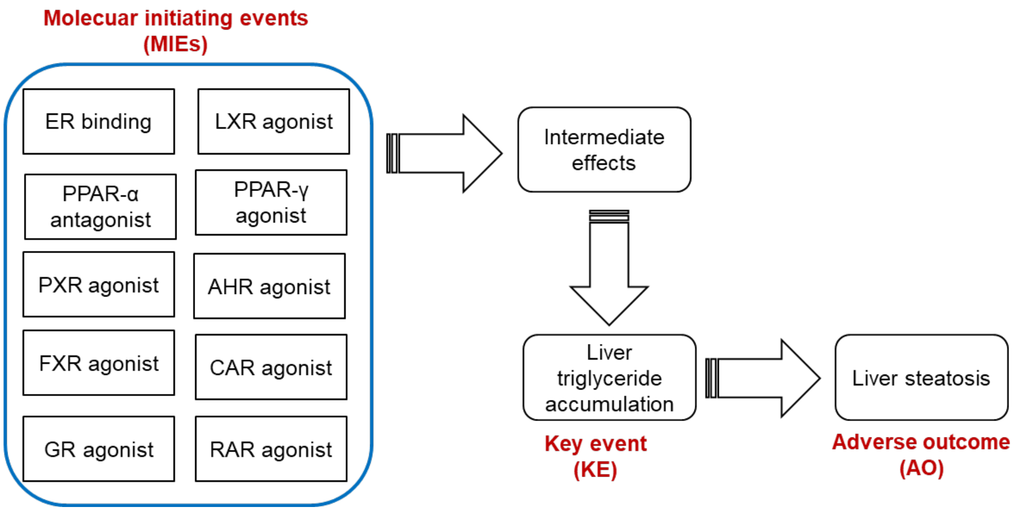

Supplementary Figure S1. Summary of previously proposed liver steatosis adverse outcome pathway

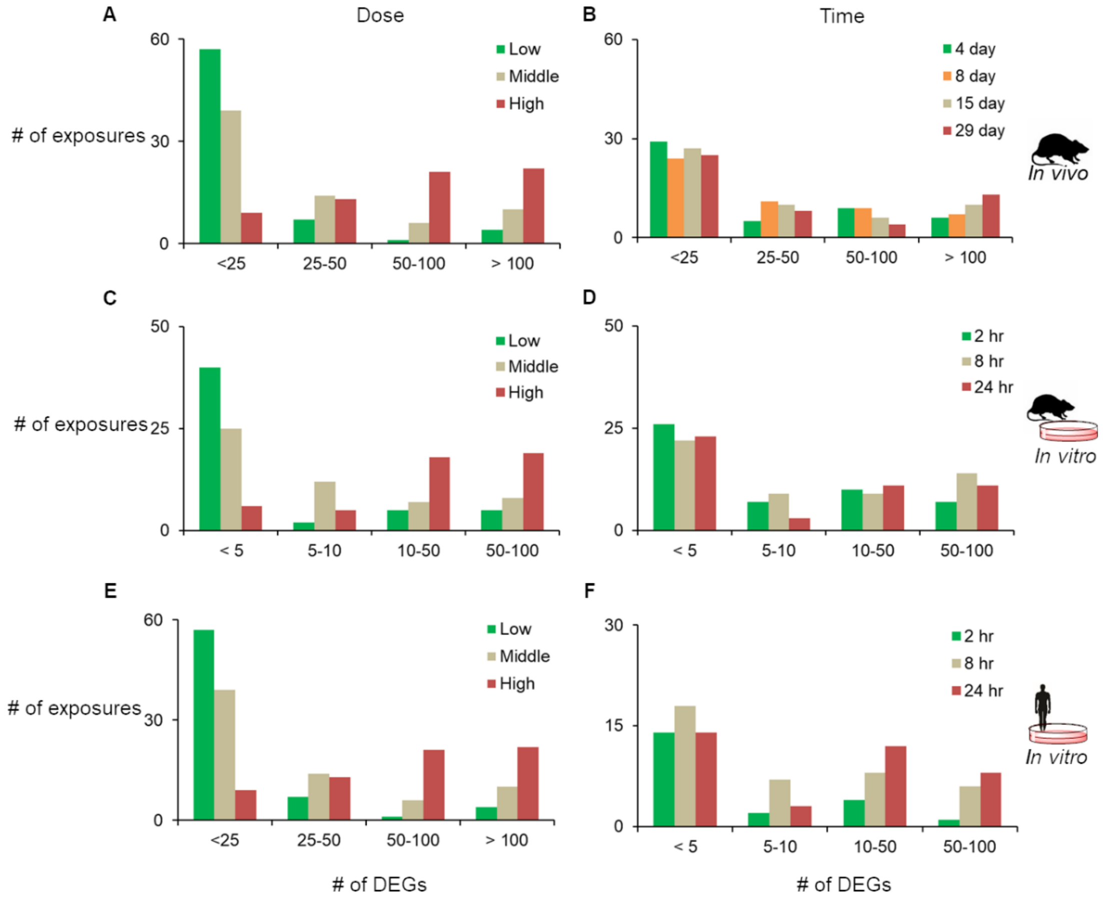

Supplementary Figure S2. Effect of dose and time on the number of differentially expressed genes (DEGs) across rat *in vivo*, rat *in vitro*, and human *in vitro* studies. Number of DEGs associated with exposures with respect to A. rat *in vivo* dose, B. rat *in vivo* duration, C. rat *in vitro* dose, D. rat *in vitro* duration, E. human *in vitro* dose, F. human *in vitro* duration.

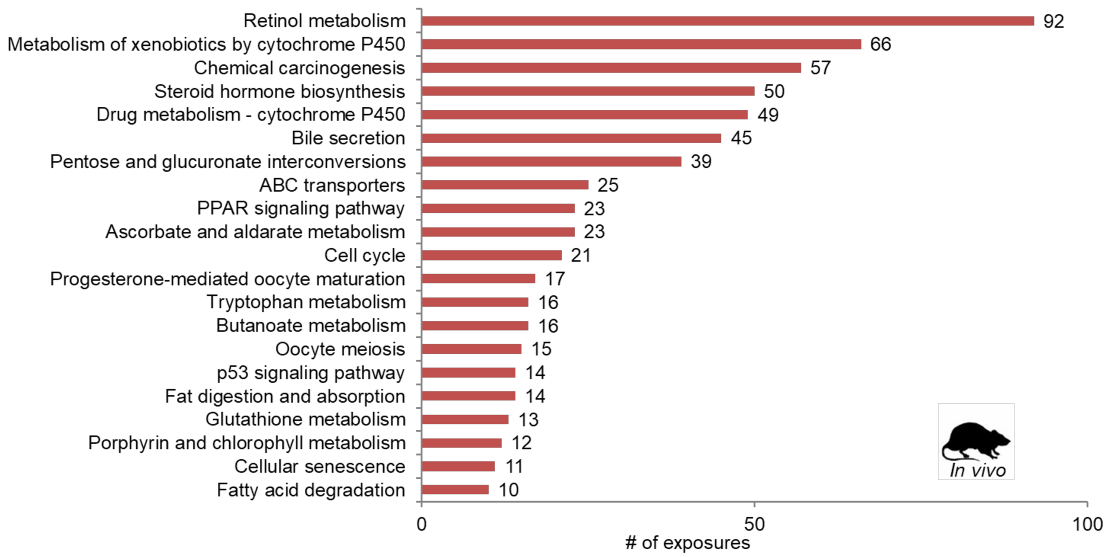

Supplementary Figure S3. Pathways frequently enriched upon exposure to a steatogenic chemical.

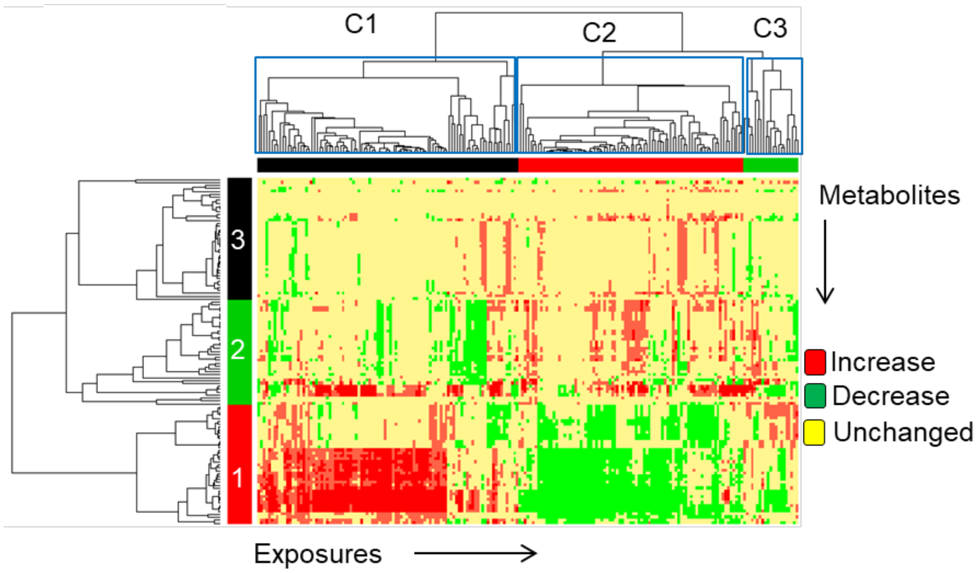

Supplementary Figure S4. Cluster analysis of predicted metabolites across 205 chemical exposure conditions (rat *in vivo* studies). 205 chemical exposures clustered into three condition clusters which were marked as C1, C2, and C3. The predicted metabolites grouped into three metabolite clusters marked as 1, 2, and 3. In the heatmap, the red color represents the metabolites predicted to increase, green color represents the metabolites that were predicted to decrease, and the yellow color represents the metabolites that were predicted to remain unchanged for different chemical exposures.

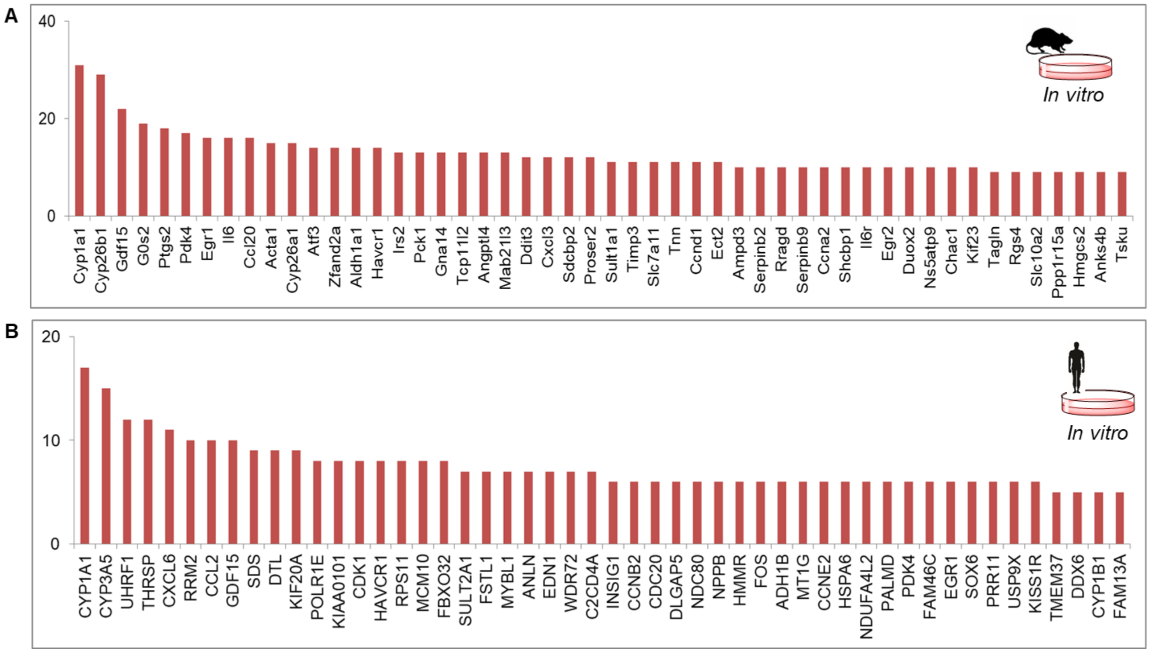

Supplementary Figure S5. The 50 most frequently observed differentially expressed genes (DEGs) across exposure conditions in A. rat *in vitro* and B. human *in vitro* studies.

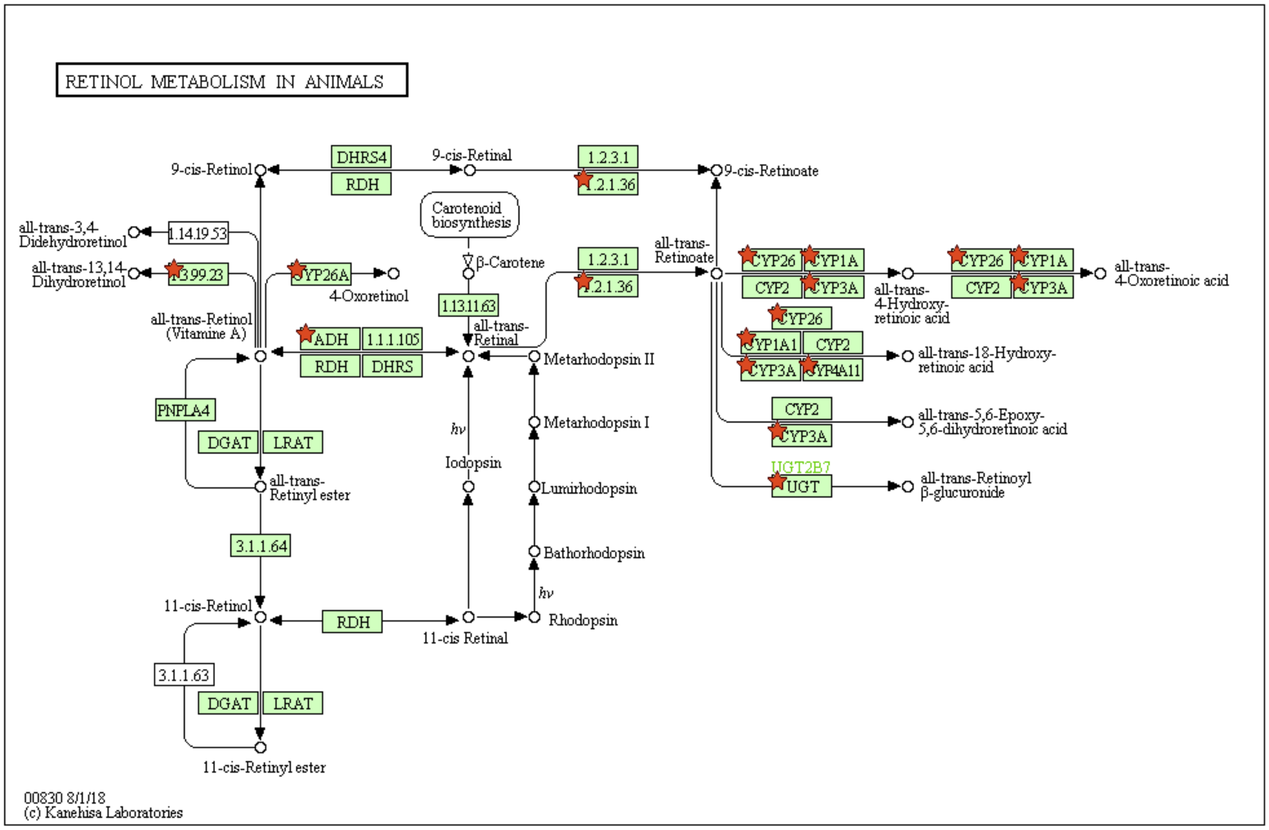

Supplementary Figure S6. Retinol metabolism pathway with frequently differentially expressed genes (DEGs) mapped on the pathway. The mapped DEGs are marked as red stars.
